# The proangiogenic effects of extracellular vesicles secreted by dental pulp stem cells derived from periodontally compromised teeth

**DOI:** 10.1186/s13287-020-01614-w

**Published:** 2020-03-06

**Authors:** Huan Zhou, Xuan Li, Yuan Yin, Xiao-Tao He, Ying An, Bei-Min Tian, Yong-Long Hong, Li-An Wu, Fa-Ming Chen

**Affiliations:** 1 State Key Laboratory of Military Stomatology, National Clinical Research Center for Oral Diseases, School of Stomatology, Fourth Military Medical University, Xi’an, 710032 Shaanxi People’s Republic of China; 2 Stomatology Center, Shenzhen Hospital of Southern Medical University, Shenzhen, Guangdong People’s Republic of China

**Keywords:** Inflammation, Periodontitis, Dental pulp stem cells, Extracellular vesicles, Angiogenesis

## Abstract

**Background:**

Although dental pulp stem cells (DPSCs) isolated from periodontally compromised teeth (P-DPSCs) have been demonstrated to retain pluripotency and regenerative potential, their use as therapeutics remains largely unexplored. In this study, we investigated the proangiogenic effects of extracellular vesicles (EVs) secreted by P-DPSCs using in vitro and in vivo testing models.

**Methods:**

Patient-matched DPSCs derived from periodontally healthy teeth (H-DPSCs) were used as the control for P-DPSCs. Conditioned media (CMs) derived from H-DPSCs and P-DPSCs (H-CM and P-CM), CMs derived from both cell types pretreated with the EV secretion blocker GW4869 (H-GW and P-GW), and EVs secreted by H-DPSCs and P-DPSCs (H-EVs and P-EVs) were prepared to test their proangiogenic effects on endothelial cells (ECs). Cell proliferation, migration, and tube formation were assessed using the Cell Counting Kit-8 (CCK-8), transwell/scratch wound healing, and Matrigel assays, respectively. Specifically, quantitative reverse transcriptase-polymerase chain reaction (qRT-PCR) and western blot analysis were used to examine the expression levels of angiogenesis-related genes/proteins in ECs in response to EV-based incubation. Finally, a full-thickness skin defect model was applied to test the effects of EVs on wound healing and new vessel formation.

**Results:**

Both H-CM and P-CM promoted EC angiogenesis, but the proangiogenic effects were compromised when ECs were incubated in H-GW and P-GW, wherein the EV secretion was blocked by pretreatment with GW4869. In EV-based incubations, although both H-EVs and P-EVs were found to enhance the angiogenesis-related activities of ECs, P-EVs exerted a more robust potential to stimulate EC proliferation, migration, and tube formation. In addition, P-EVs led to higher expression levels of angiogenesis-related genes/proteins in ECs than H-EVs. Similarly, both P-EVs and H-EVs were found to accelerate wound healing and promote vascularization across skin defects in mice, but wounds treated with P-EVs resulted in a quicker healing outcome and enhanced new vessel formation.

**Conclusions:**

The findings of the present study provide additional evidence that P-DPSCs derived from periodontally diseased teeth represent a potential source of cells for research and therapeutic use. Particularly, the proangiogenic effects of P-EVs suggest that P-DPSCs may be used to promote new vessel formation in cellular therapy and regenerative medicine.

## Background

Dental pulp stem cells (DPSCs) are mesenchymal stem cell (MSC)-like populations that can be noninvasively harvested from pulp tissue of extracted human teeth; hence, these cells can be relatively easily obtained and are easy to access for use (reviewed in [1]). Due to their robust self-renewal capacity and pluripotency, DPSCs have paved the way for not only regenerating tooth-related tissues such as dental pulp [2] and the periodontium [3] but also repairing other types of tissue insults such as bone defects [4] and central nervous system damage [5]. Although accumulating evidence suggests that MSCs derived from bone marrow (BMMSCs), adipose tissue (ADSCs), and dental pulp (DPSCs) are all promising MSC types for regenerative purposes, DPSCs, at least in certain situations, were found to contain a higher percentage of stem/progenitor cells and to exhibit higher proliferation and osteogenic potential than donor-matched BMMSCs [6]. In addition, DPSCs have shown superior resistance to subculture, cryopreservation [7], and inflammation-induced senescence [8]. Taken together, these findings indicate that DPSCs are highly likely to maintain their multidifferentiation and regenerative potential within an inflamed microenvironment and thus would be suitable for use in combating inflammation-induced disease.

Although DPSCs can be isolated from exfoliated deciduous teeth, extracted wisdom teeth, and sometimes teeth extracted for orthodontic reasons [2, 5], we still cannot ensure that a patient will have a tooth available for cell isolation when cell therapy is needed. In this context, stem cells from allogeneic tissues might be an alternative choice, but the use of allogeneic MSCs adds additional safety and ethical concerns [9]. In view of this, scientists have sought to use DPSCs derived from inflamed pulp tissues, as these tissues are more easily accessible in clinics and often can be obtained from teeth with crown fractures [10], irreversible pulpitis [11–13], and periodontitis [14, 15]. Particularly, pulp tissues within periodontally compromised teeth can be used for the production of DPSCs because these cells, to a certain degree, have been demonstrated to maintain their pluripotency and regenerative potential provided that pulp vitality is not totally lost [14, 15]. Previously, we successfully isolated DPSCs from teeth that were extracted teeth due to severe periodontitis (P-DPSCs); their pluripotential capacity has been demonstrated using in vitro and in vivo models, suggesting that these traditionally discarded teeth may be an inexhaustible cell source for research and therapeutic purposes [14]. Although P-DPSCs are becoming relevant in cell production, how to use these cells for therapeutic purposes requires further exploration. Considering that the most direct use of DPSCs is pulp regeneration and that a prerequisite for cells to regenerate functional pulp is their proangiogenic effects for inducing vasculature formation, we herein investigated the angiogenic potential and vessel formation ability of P-DPSCs using in vitro and in vivo testing models.

Based on current understanding, the beneficial proangiogenic effects of stem cells in cellular therapy are most likely mediated via their paracrine mechanism (reviewed in [16]). It is now well recognized that stem cells can release an abundant mixture of cytokines, growth factors, chemokines, and extracellular vesicles (EVs), all of which have been demonstrated to contain functional elements that are useful for tissue regeneration. In particular, EVs within these secreted elements carry a complex cargo including but not limited to various mRNAs, microRNAs, and a spectrum of anti-apoptotic and proangiogenic factors; these agents are known to be the main mediators contributing to cell paracrine effects (reviewed in [17]). In this context, EVs derived from DPSCs have been demonstrated to trigger regeneration of dental pulp-like tissue [18] and indeed exhibit the capacity to promote angiogenesis in vitro and in vivo [19]. Therefore, the secreted elements contained within conditioned media (CMs) derived from P-DPSCs, particularly their secreted EVs, can be applied to investigate the proangiogenic effects of P-DPSCs.

In this study, we produced CMs using patient-matched DPSCs derived from periodontally healthy teeth (H-DPSCs) and P-DPSCs (H-CM and P-CM), CMs derived from both cells pretreated by GW4869 (EV secretion inhibited; H-GW and P-GW), and isolated EVs secreted by H-DPSCs and P-DPSCs (H-EVs and P-EVs); these cellular materials were used in parallel to test their proangiogenic effects on endothelial cells (ECs). Finally, the ability of EVs (H-EVs and P-EVs) to improve wound healing and promote new vessel formation was tested in a full-thickness skin defect model. This is the first endeavor to explore the potential of using P-DPSCs for angiogenesis and new vessel formation in cellular therapy and regenerative medicine.

## Methods

### Isolation and identification of H-DPSCs and P-DPSCs

Patients visiting the Stomatological Hospital of Fourth Military Medical University (FMMU) who had (i) at least one tooth with full or partial pulp vitality that was to be extracted due to irreversible periodontitis and (ii) at least one other periodontally healthy tooth that was to be extracted due to nonfunctional or impacted reasons (often the third molars) were asked to donate their teeth for cell isolation and related research. The experimental protocol was approved by the Ethics Committee of the Stomatological Hospital of FMMU (201203), and informed consent was signed by all the subjects. Finally, 5 pairs of teeth were successfully obtained from 5 systemically healthy donors (male. 2; female. 3; age. 24~41 years); dental pulp tissues from periodontally healthy teeth (*n* = 5) and periodontitis teeth (*n* = 6) were used to isolate H-DPSCs and P-DPSCs in parallel according to our previously reported methods [14]. Briefly, the teeth were rinsed with adequate phosphate-buffered saline (PBS; Corning, NY, USA) before sawing to expose the pulp cavity. Then, the pulp tissues were removed from the teeth and cut into small pieces (0.5–1 cm in length). After that, the tissue tips were immersed in digestive solution (3 mg/mL collagenase type I; DIYIBio; Shanghai, China) and incubated for 1 h at 37 °C (with vigorous shaking every 10 min), followed by transfer into 12-well plates (Corning, Lowell, MA, USA). The primary cells were cultured in α-minimum essential medium (α-MEM; Gibco BRL, Grand Island, NY, USA) containing 10% fetal bovine serum (FBS; Sijiqing, Hangzhou, China) and 1% penicillin-G/streptomycin (Invitrogen, Carlsbad, CA, USA) at an atmosphere of 5% CO_2_ at 37 °C and passaged after reaching 80–90% confluence. The limiting dilution technique was applied for DPSC purification. DPSCs at passages P3-P5 were characterized by a colony-forming assay, a proliferation assay, flow cytometry, and a multiple differentiation assay and then used for subsequent experiments.

For the colony-forming assay, DPSCs (P3) from both groups were seeded into 100-mm-diameter culture dishes (Corning) with complete α-MEM at a density of 1 × 10^3^ cells/dish. The culture medium was refreshed every 3 days. After incubation for 14 days, DPSCs were fixed with 4% paraformaldehyde (Servicebio, Wuhan, China) for 30 min, stained with crystal violet (Heart, Xi’an, China) for 30 min and washed with distilled water three times. Then, the number of colonies was counted with an inverted microscope (Olympus, Tokyo, Japan), and only aggregates consisting of 50 or more cells were termed colonies.

A Cell Counting Kit-8 (CCK-8) assay was utilized to measure the proliferation ability of DPSCs. In brief, cells (P3) were seeded into 96-well plates (Corning) with complete α-MEM at a density of 2 × 10^3^ cells per well (four replicates per group). Ten microliters of CCK-8 reagent (Dojindo, Shanghai, China) was added to each well at a fixed time each day during the 7-day culture, and the cells were incubated for another 3 h at 37 °C. The optical density (OD) value was recorded at 450 nm by an Infinite M200 PRO microplate reader (TECAN, Männedorf, Switzerland).

A flow cytometry assay was applied to identify the cell immunophenotypes. Briefly, DPSCs (P3) were collected and washed twice with PBS containing 3% FBS, and then, the cell suspension was divided into sterile Eppendorf tubes (Axygen, Tewksbury, MA, USA) and incubated with monoclonal antibodies against human CD90, CD105, CD146, CD34, CD45, or CD31 (all from eBioscience, San Diego, CA, USA) for 1 h at 4 °C in the dark. Cell suspensions incubated with PBS served as a negative control. Next, DPSCs were washed twice with PBS to remove excessive antibodies and then resuspended in 400 μL PBS, followed by analysis with a Beckman Coulter Epics XL cytometer (Beckman Coulter, Fullerton, CA, USA).

To identify multiple differentiation potential, DPSCs (P4) were seeded into 6-well plates and cultured in complete α-MEM at a density of 2 × 10^5^ cells per well. After the DPSCs reached 80% confluence, their culture medium was changed to osteogenic differentiation medium, adipogenic differentiation medium, or chondrogenic differentiation medium (all from Cyagen, Guangzhou, China). After induction for 21 days (for osteogenic differentiation or adipogenic differentiation) or 28 days (for chondrogenic differentiation), differentiated cells were fixed with 4% paraformaldehyde and then separately subjected to Alizarin Red S staining (for osteogenic differentiation), Oil Red O staining (for adipogenic differentiation), and Alcian blue staining (for chondrogenic differentiation).

### Conditioned medium (CM)-based incubation of ECs

#### Preparation of CMs derived from H-DPSCs and P-DPSCs

H-DPSC- and P-DPSC-derived CMs were prepared in parallel based on the methods reported previously [20]. Briefly, DPSCs (H-DPSCs and P-DPSCs) were cultured with complete α-MEM and incubated until they reached approximately 90% confluence. Then, the cells were washed three times with PBS prior to culturing with serum-free α-MEM media. After another incubation for 48 h, the cell-derived supernatant was harvested, centrifuged at 3000×*g* for 3 min and 1500×*g* for 5 min, filtered with 0.22-μm filters (Millipore, Billerica, MA, USA) and stored at − 80 °C for further experiments. Cell-derived supernatants were also prepared from cells that were pretreated with culture media containing GW4869 (10 μM; Sigma-Aldrich, St. Louis, USA) for 12 h before culturing in serum-free α-MEM media, wherein GW4869 was used for the inhibition of EV secretion. Prior to use for EC culture, the supernatants derived from H-DPSCs, GW4869-pretreated H-DPSCs, P-DPSCs, and GW4869-pretreated P-DPSCs were mixed with endothelial growth medium-2 (EGM-2; Lonza, Walkersville, MD, USA) at a ratio of 1:1 and termed H-CM, H-GW, P-CM, or P-GW. Cell proliferation, migration, and tube formation of ECs in response to incubation with various CMs were evaluated using established in vitro models, wherein a mixture of fresh α-MEM medium and EGM-2 was used as a blank control for H-CM and P-CM.

#### Proliferation assay

Human umbilical vein ECs were purchased from the American Type Culture Collection (ATCC; CRL-1730) and seeded in 96-well plates (Corning) at a concentration of 5 × 10^3^ cells/well. Following culturing in various CMs (four replicates per CM), CCK-8-based cell proliferation assays (across a 5-day period) were performed as described previously in this manuscript.

#### Cell migration assay

Transwell and scratch wound healing assays were used to evaluate cell migration in response to various CM-based incubations. For the transwell assay, ECs (2 × 10^4^ cells/well) suspended in 100 μL serum-free medium were seeded into the upper chamber of 24-well transwell culture plates (8-μm pore-sized filters; Corning), while 500 μL CM (H-CM, P-CM, H-GW, or P-GW), or the established control medium for H-CM and P-CM, was added to the lower chamber. After 8 h of incubation, nonmigrated cells on the upper surface of inserts were removed gently with a cotton swab, and then, the cells that migrated to the lower surface of membranes were fixed with 4% paraformaldehyde prior to staining with crystal violet (Heart) for 30 min. Images were captured, and the number of migrated cells was counted in five randomly selected fields per well. For the scratch wound healing assay, ECs (4 × 10^5^ cells/well) were seeded into 6-well plates (Corning). After the cells reached 90% confluence, one scratch was made in each well with a sterile 1 mL pipette tip, and then, the floating cells were removed by washing twice with PBS. After that, ECs were exposed to the various CMs. Scratch images were obtained at baseline (0 h) and at different observation time points (12 h; 24 h), and the outcomes were analyzed with ImageJ software.

#### Tube formation assay

A Matrigel tube formation assay was conducted to investigate network formation of ECs by the method described previously [21]. In brief, 96-well plates were coated with 50 μL cold Matrigel (Corning; #356234) and incubated at 37 °C for 30 min. Then, 110 μL different CMs containing 2 × 10^4^ ECs were added to each well. After incubation for 6 h, the images of tube formation were observed and photographed. Indicators of tube formation ability, including covered area, total branching points, total tube length, and total loops, were analyzed with ImageJ software in five randomly chosen fields.

### Production of EVs derived from H-DPSCs and P-DPSCs (H-EVs and P-EVs)

For extraction of EVs, the medium of H-DPSCs or P-DPSCs was collected after 48 h of culture, and the centrifugation steps were performed as described previously [22]. Briefly, the harvested medium was centrifuged at 300×*g* for 10 min and 2000×*g* for 10 min to eliminate cells. Then, the obtained supernatant was centrifuged at 10,000×*g* for 30 min to eliminate cellular debris. After that, the following supernatant was centrifuged at 100,000×*g* for 70 min and additionally washed with PBS at 100,000×*g* for 70 min (Ultracentrifuge, Beckman Coulter). Finally, the supernatant was discarded, and EV pellets were resuspended in PBS for further experiments or cryopreserved at − 80 °C. The morphologies of EVs were observed with transmission electron microscopy (Hitachi, Tokyo, Japan), the concentration and size of EVs were measured and analyzed with nanoparticle tracking analysis (NanoSight 300; Malvern Instruments, Malvern, UK), and biomarker proteins for EVs were detected by the western blot assay (see the “Western blot analysis” section). To examine the incorporation of EVs, H-EVs and P-EVs were labeled with PKH67 (Sigma-Aldrich, St. Louis, MO, USA) according to the protocol. Briefly, EVs were resuspended in diluent C, mixed with PKH67, and incubated for 4 min. Then, 5% bovine serum albumin (BSA; heart) was added to neutralize the reaction. Afterwards, the labeled EVs were washed in PBS at 100,000×*g* for 70 min and then incubated with ECs at 37 °C for 4 h. 4′,6-diamidino-2-phenylindole (DAPI; Heart) was used to label the nuclei. Images of EV uptake were obtained with a confocal imaging system (Carl Zeiss, Oberkochen, Germany).

### EV-based incubation of ECs

Similar to CM-based incubation, cell proliferation, migration, and tube formation of ECs in response to incubation with various EVs were determined, wherein the medium was changed to endothelial basal medium (EBM-2; Lonza) containing 5% FBS in the presence of 100 μg/mL H-EVs (H-EVs group) or 100 μg/mL P-EVs (P-EVs group); the same amount of PBS was used as a control (control group). Quantitative real-time PCR (qRT-PCR) and western blot analysis were applied to evaluate the cellular responses at the gene and protein levels.

#### qRT-PCR analysis

For gene expression analysis, cell samples were washed twice with PBS. After that, total RNA was extracted with a MiniBEST Universal RNA Extraction Kit (Takara, Tokyo, Japan) and then reverse transcribed to cDNA using the PrimeScript™ RT reagent Kit (Takara). qRT-PCR analysis with TB Green™ Premix Ex Taq™ II (Takara) was conducted and analyzed using a Real-Time PCR Detection System (Bio-Rad, Hercules, CA, USA). β-Actin was employed to normalize the levels of the genes of interest. Primer sequences used for qRT-PCR were as follows: β-actin: forward, 5′-TGGCACCCAGCACAATGAA-3′, and reverse, 5′-CTAAGTCATAGTCCGCCTAGAAGCA-3′; h-VEGF: forward, 5′- CATCCAATCGAGACCCTGGTG-3′, and reverse, 5′-TTGGTGAGGTTTGATCCGCATA-3′; and h-AngII: forward, 5′-GCTGAAGTATTCAAATCAGGACACA-3′, and reverse, 5′-ATCAACGCTGCCATCCTCA-3.

#### Western blot analysis

The protein extracts of cells (or EVs, see the “Production of EVs derived from H-DPSCs and P-DPSCs (H-EVs and P-EVs)” section) were subjected to sodium dodecyl sulfate-polyacrylamide gel electrophoresis (SDS-PAGE) and transferred to PVDF membranes (Millipore, Billerica, MA, USA). Then, the membranes were blocked in 5% nonfat milk in Tris-buffered saline Tween-20 (TBST, Heart). After incubating with primary antibodies including anti-β-actin (1:1000; Proteintech, Rosemont, USA; #60008-1-lg), anti-ALIX (1:1000; Cell Signaling Technology, Danvers, MA, USA; #2171), anti-HSP-70 (1:1000; Cell Signaling Technology; #4876), anti-CD9 (1:1000; Cell Signaling Technology; #13174), anti-CD81 (1:1000; Abcam, Cambridge, Britain; ab109201), anti-VEGF(1:1000; Abcam; ab46154), and anti-AngII (1:100; Santa Cruz, CA, USA; sc-74,403) at 4 °C overnight, the membranes were washed three times with TBST. Then, the cells were incubated with horseradish peroxidase-conjugated secondary antibodies (1:5000, Proteintech; SA00001-1 or SA00001-2) for 2 h at room temperature. Subsequently, blots were detected using chemiluminescent detection reagent (Zeta Life, CA, USA), and the protein bands were analyzed with ImageJ software. β-actin was employed as the housekeeping gene for internal normalization.

### Use of EVs in a skin wound healing model

#### Mouse skin wound model and treatments

Thirty male C57BL/6 mice (8 weeks old, weighing 20–25 g; purchased from the Animal Research Committee of FMMU) were used in this study, and all procedures were approved by the Animal Research Committee of FMMU. A full-thickness excisional skin wound was created on the dorsum of each mouse as previously reported [23]. Then, the mice were randomly divided into three groups (10 mice per group): an H-EV group, a P-EV group, and a control group. Mice in each group were designated to receive subcutaneous injection of either H-EVs (200 μg in 100 μL PBS), P-EVs (200 μg in 100 μL PBS) or 100 μL of PBS only (4 sites around the wounds; 25 μL per site). To evaluate the rate of wound healing, wounds were photographed and measured with a metric ruler at baseline and 4, 9, and 14 days after treatment, and the wound area was analyzed with ImageJ software. The undersurface of the skin was observed by a microscope (Olympus) to detect newly formed vessels on day 14 after the operation.

#### Histological and immunofluorescence analysis

Vascularization is essential to wound healing and tissue repair (reviewed in [24]). Thus, histological analysis and immunohistochemistry staining for angiogenesis markers were used to evaluate wound closure as well as newly formed vessels. Two weeks after surgery, mice were sacrificed, and the skin specimens were harvested and analyzed according to the methods reported previously [23]. Briefly, the collected skin tissues (including the wound healing and surrounding healthy skin) were fixed with 4% paraformaldehyde solution, dehydrated with ethanol, and embedded in paraffin. Then, the specimens were cut into 10-μm-thick sections and subjected to hematoxylin and eosin (H&E) staining. In addition, immunofluorescence staining for VEGF and CD31 was performed to estimate the newly formed capillaries, as previously reported [25]. In brief, the obtained samples were fixed with 4% paraformaldehyde overnight at 4 °C, dehydrated in 30% sucrose solution, and then embedded. Then, the 10-μm-thick sections were incubated with primary antibodies, including anti-VEGF (1:100, Abcam; ab2349) and anti-CD31 (1:100, Abcam; ab9498), overnight at 4 °C and then incubated with secondary antibodies (1:300 or 1:400, Servicebio; GB21303 or GB25303) for 1 h at room temperature. Images were captured and analyzed with Image-Pro Plus 6 software.

### Statistical analysis

All assays were performed at least three times independently, and the data are presented as the mean ± standard deviation (S.D.). Differences between groups were assessed by the paired *t* test (for two-group comparisons) and one-way analysis of variance (ANOVA) followed by Tukey’s posttest (for multiple groups). A level of *p* < 0.05 was judged to be statistically significant.

## Results

### Characterization of H-DPSCs and P-DPSCs

**Fig. 1:**
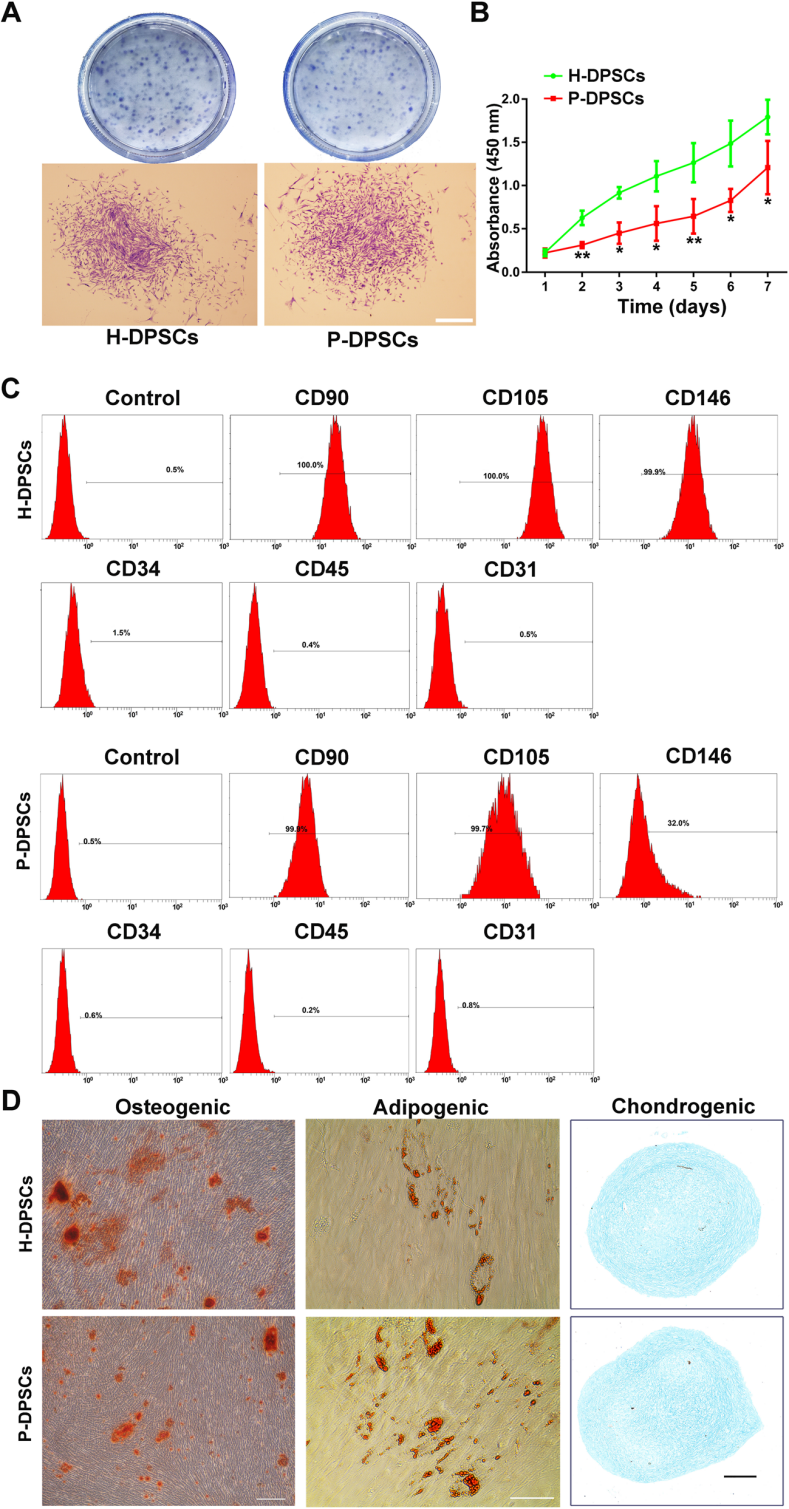
Isolation and identification of H-DPSCs and P-DPSCs. **a** Representative images of colony units in a general view (first row of images) and a single colony of H-DPSCs and P-DPSCs observed by microscopy (second row of images; scale bar, 200 μm). **b** Proliferative activity of H-DPSCs and P-DPSCs evaluated by CCK-8 assay (*n* = 3; *P < 0.05, **P < 0.01 vs. the H-DPSC group). **c** Surface markers of H-DPSCs and P-DPSCs assayed by flow cytometry. **d** Multiple differentiation potentials of H-DPSCs and P-DPSCs: Alizarin Red S staining for osteogenic differentiation (left; scale bar, 200 μm), Oil Red O staining for adipogenic differentiation (middle; scale bar, 200 μm), and Alcian blue staining for chondrogenic differentiation (right; scale bar, 200 μm)

H-DPSCs and P-DPSCs were successfully isolated from pulp tissues of 11 teeth (periodontally healthy teeth, 5; periodontitis teeth, 6). Five teeth belonging to 2 donors were excluded due to culture failure (if cell isolation failed for one tooth, the participant was excluded). Almost all of the cells initially grew from pulp tissues after incubation for 7 days (Fig. S1a) and exhibited clone-like growth as well as a spindle-shaped appearance (Fig. S1b). It was apparent that DPSCs in both groups presented colony-forming ability (Fig. 1a). In terms of the CCK-8 assay, H-DPSCs displayed a significantly higher proliferative rate than P-DPSCs (*P* < 0.05 or *P* < 0.01; Fig. 1b). Flow cytometry assays were conducted to detect the surface markers of DPSCs. Notably, both P-DPSCs and H-DPSCs were positive for MSC markers, including CD90, CD105, and CD146, and negative for hematopoietic cell markers, such as CD31, CD34, and CD45 (Fig. 1c). In addition, both P-DPSCs and H-DPSCs could readily be induced to differentiate into osteogenic, adipogenic, and chondrogenic lineages when cultured in their respective differentiation media, as evidenced by Alizarin Red S staining, Oil Red O staining, and Alcian blue staining, respectively (Fig. 1d).

### Cellular responses of ECs to incubation with various CMs

#### P-CM enhanced the angiogenic activities of ECs in vitro

**Fig. 2:**
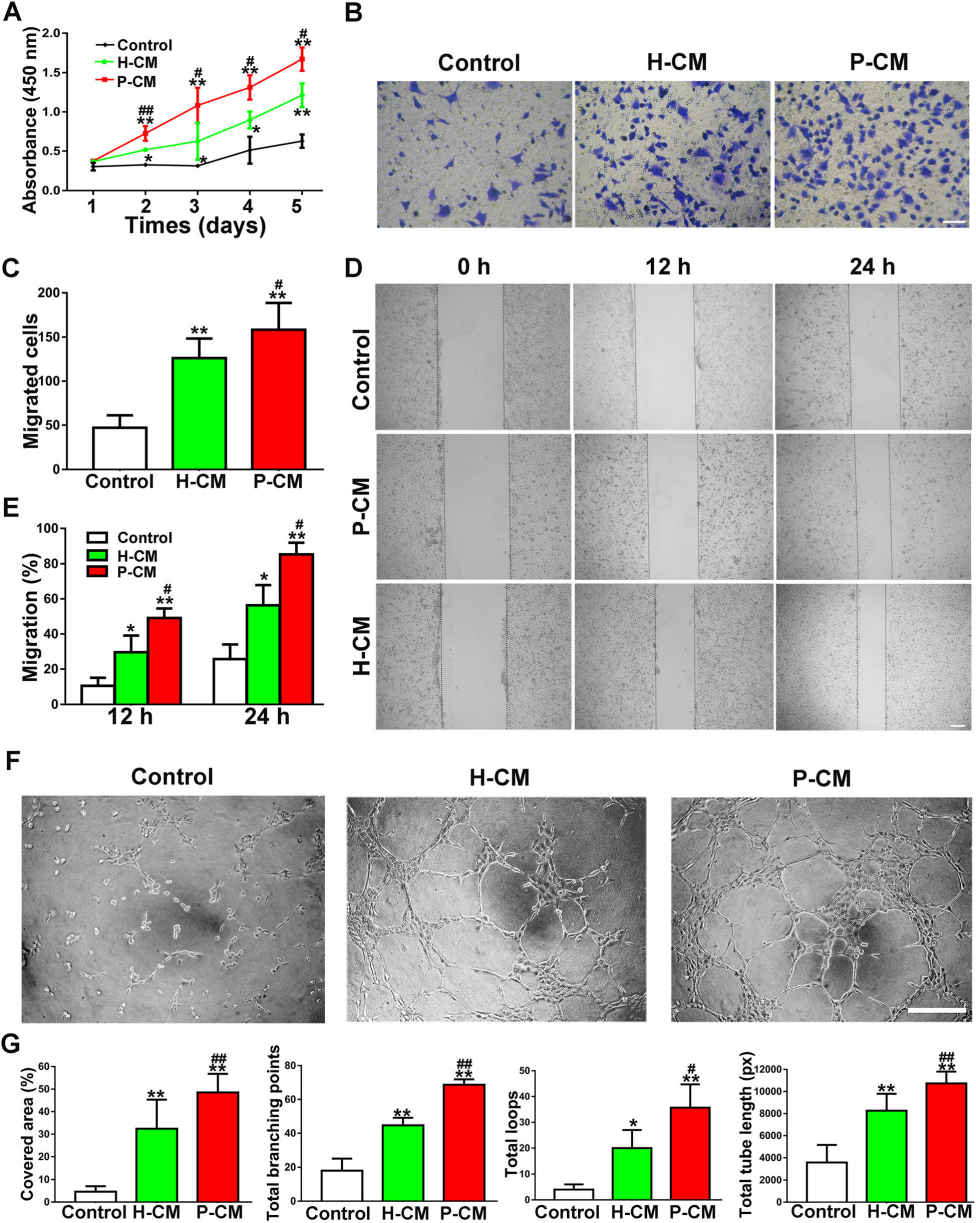
H-CM and P-CM enhanced the angiogenic activities of ECs. **a** The proliferation of ECs exposed to H-CM and P-CM was tested by CCK-8 assay (*n* = 3). **b** The migration of ECs stimulated by H-CM and P-CM was detected by transwell assay (scale bar, 100 μm). **c** Quantitative analysis of the migrated cells in **b** (*n* = 5). **d** Representative images of the scratch wound assay of ECs treated with H-CM and P-CM (scale bar, 200 μm). **e** Quantitative analysis of the migration rates in **d** (*n* = 5). **f** Representative images of the tube formation assay in ECs treated with H-CM and P-CM (scale bar, 200 μm). **g** Quantitative analyses of the covered area, total branching points, total loops, and total tube length in **f** (*n* = 3). *P < 0.05, **P < 0.01 vs. the control group; ^**#**^*P* < 0.05, ^**##**^*P* < 0.01 vs. the H-CM group

It is well recognized that paracrine activity contributes considerably to MSC-based therapy [26]. To analyze the paracrine effects of DPSCs, ECs were exposed to H-CM and P-CM, and angiogenic activities related to proliferation, migration, and tube formation were analyzed. As shown in Fig. 2a, both H-CM and P-CM significantly enhanced EC proliferation compared with the control (*P* < 0.05 or *P* < 0.01); additionally, the proliferation rate was higher in the P-CM group than in the H-CM group (*P* < 0.05 or *P* < 0.01). Similar trends were observed in the transwell migration assay (*P* < 0.05 or *P* < 0.01; Fig. 2b, c) and scratch wound healing assay (*P* < 0.05 or *P* < 0.01; Fig. 2d, e). In addition, tube formation assays revealed that incubation with either P-CM or H-CM could promote angiogenesis when compared with that in the control group (Fig. 2f). With the exception of the covered area (*P* > 0.05), ECs incubated with P-CM demonstrated a stronger tube formation ability than those incubated with H-CM in terms of quantitative analyses of the total branching points (*P* < 0.01), total loop numbers (*P* < 0.05), and total tube length (*P* < 0.01) (Fig. 2g).

#### Inhibition of EV secretion impaired CM-mediated angiogenesis

**Fig. 3:**
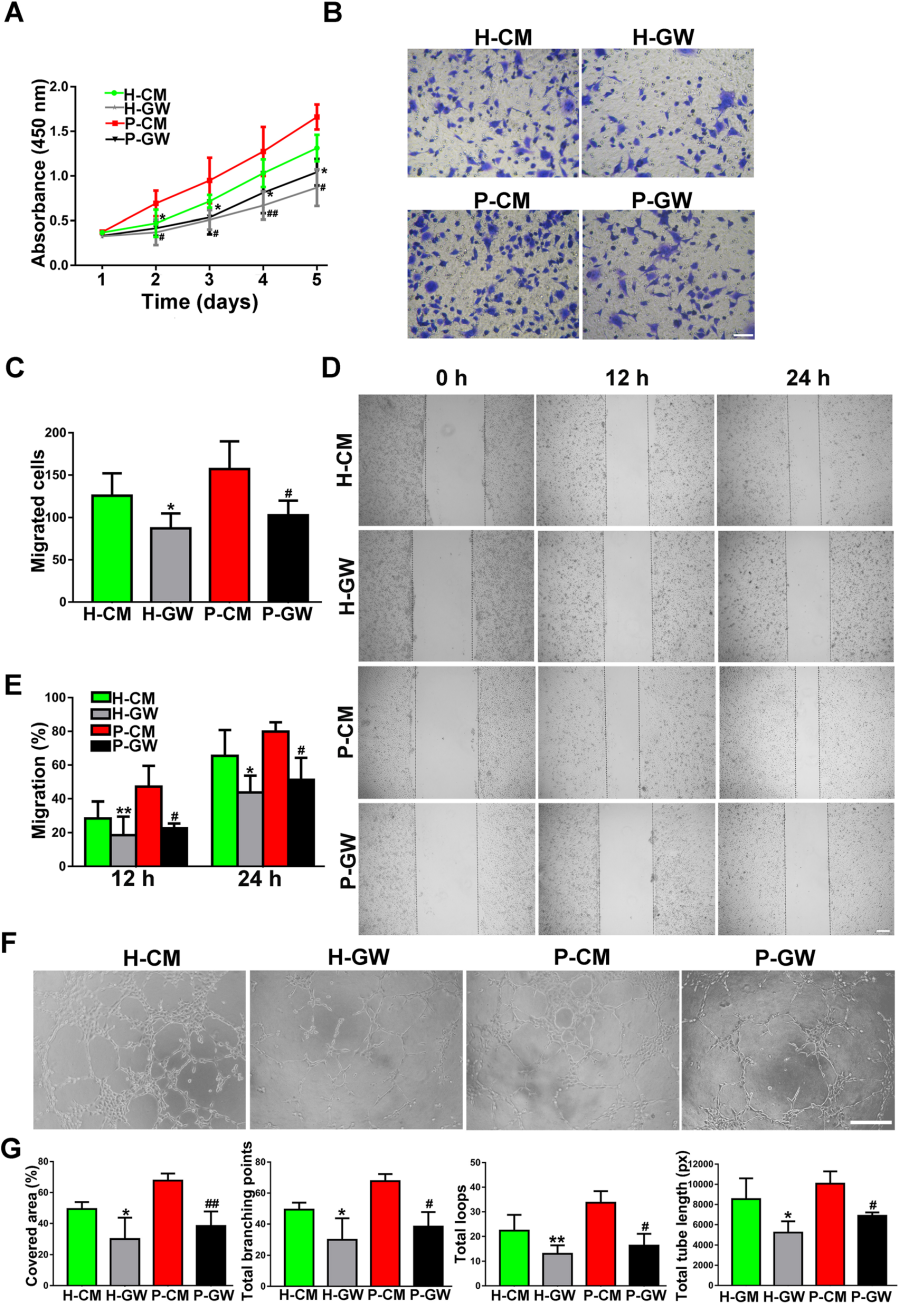
EVs play a pivotal role in CM-mediated angiogenesis. **a** The proliferation of ECs exposed to H-CM, H-GW, P-CM, and P-GW was tested by CCK-8 assay (*n* = 3). **b** The migration of ECs stimulated by H-CM, H-GW, P-CM, and P-GW was detected by transwell assay (scale bar, 100 μm). **c** Quantitative analysis of the migrated cells in **b** (*n* = 3). **d** Representative images of the scratch wound assay of ECs treated with H-CM, H-GW, P-CM, and P-GW (scale bar, 200 μm). **e** Quantitative analysis of the migration rates in **d** (*n* = 3). **f** Representative images of tube formation in ECs treated with H-CM, H-GW, P-CM, and P-GW (scale bar, 200 μm). **g** Quantitative analyses of the covered area, total branching points, total loops, and total tube length in **f** (*n* = 3). *P < 0.05, **P < 0.01 vs. the H-CM group; ^**#**^*P* < 0.05, ^**##**^*P* < 0.01 vs. the P-CM group

Currently, EVs are considered to be a vital paracrine product of MSCs [27]. If EVs contribute to the proangiogenic effects of DPSCs, then inhibition of EV release would be expected to abolish or reduce the function. To verify this hypothesis, we pretreated H-DPSCs and P-DPSCs with GW4869 (a reversible blocker of neutral sphingomyelinase that controls EV secretion) [28]. It was observed that 10 μM GW4869 successfully decreased EV release by H-DPSCs and P-DPSCs (Fig. S2b) without affecting cell growth (Fig. S2a). Indeed, the increased proliferation trend of ECs exposed to H-CM and P-CM was partially inhibited by GW4869 pretreatment, as a CCK-8 assay revealed that both cells exposed to H-GW and P-GW had a lower proliferation rate than cells exposed to H-CM or P-CM (*P* < 0.05 or *P* < 0.01; Fig. 3a), respectively. In addition, the increased migration abilities of ECs stimulated by H-CM and P-CM were also attenuated by GW4869 pretreatment, with fewer migrated cells quantified by transwell migration (*P* < 0.05; Fig. 3b, c) and larger scratch wounds in the scratch wound assay (*P* < 0.05 or *P* < 0.01; Fig. 3d, e). Notably, H-GW and I-GW partially reversed the enhanced tube formation trend by H-CM and P-CM, as quantified by the capillary structure formations of ECs (*P* < 0.05 or *P* < 0.01; Fig. 3f, g).

### Identification of H-EVs and P-EVs

**Fig. 4:**
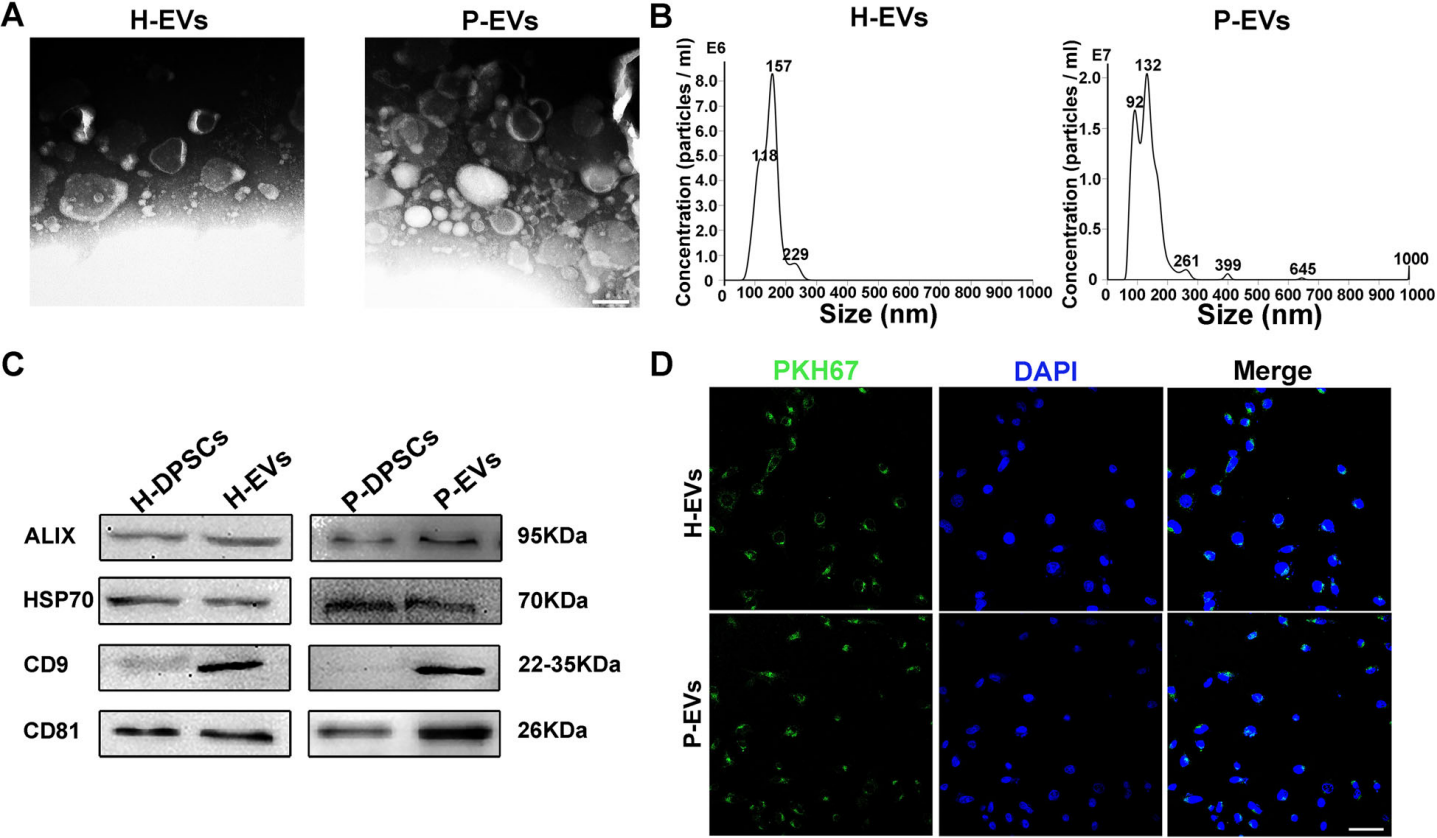
Identification and internalization of H-EVs and P-EVs. **a** Morphology of EVs under transmission electron microscopy (scale bar, 100 nm). **b** The size and concentration of EVs were identified by nanoparticle analysis. **c** EV surface markers of ALIX, HSP70, CD9, and CD81 detected by western blot. **d** Fluorescence microscopy analysis of PKH67-labeled EVs (green) internalized by ECs. Nuclei were stained with DAPI (blue) for counterstaining (scale bar, 50 μm)

Given that EVs played a crucial role in the paracrine function-mediated angiogenesis of DPSCs, H-EVs and P-EVs were isolated for further study. As expected, nanoparticles purified from the supernatants were both bilayer membrane vesicles (Fig. 4a), coinciding with the general characteristics of EVs, and nanoparticle analysis showed that the EV diameters mostly ranged from 30 to 200 nm in both groups (Fig. 4b). Additionally, western blot analysis revealed that both H-EVs and P-EVs expressed EV molecular markers, including ALIX, HSP70, CD9, and CD81 (Fig. 4c), further confirming their EV identity. Subsequently, to confirm cellular EV uptake, PKH67-labeled H-EVs and P-EVs were cocultured with ECs. After incubating for 4 h, ECs were observed to stain positive for PKH67 in both groups, confirming EV uptake (Fig. 4d).

### Cellular responses of ECs to H-EV/P-EV-based incubation

#### P-EVs promoted the proliferation and migration of ECs in vitro

**Fig. 5:**
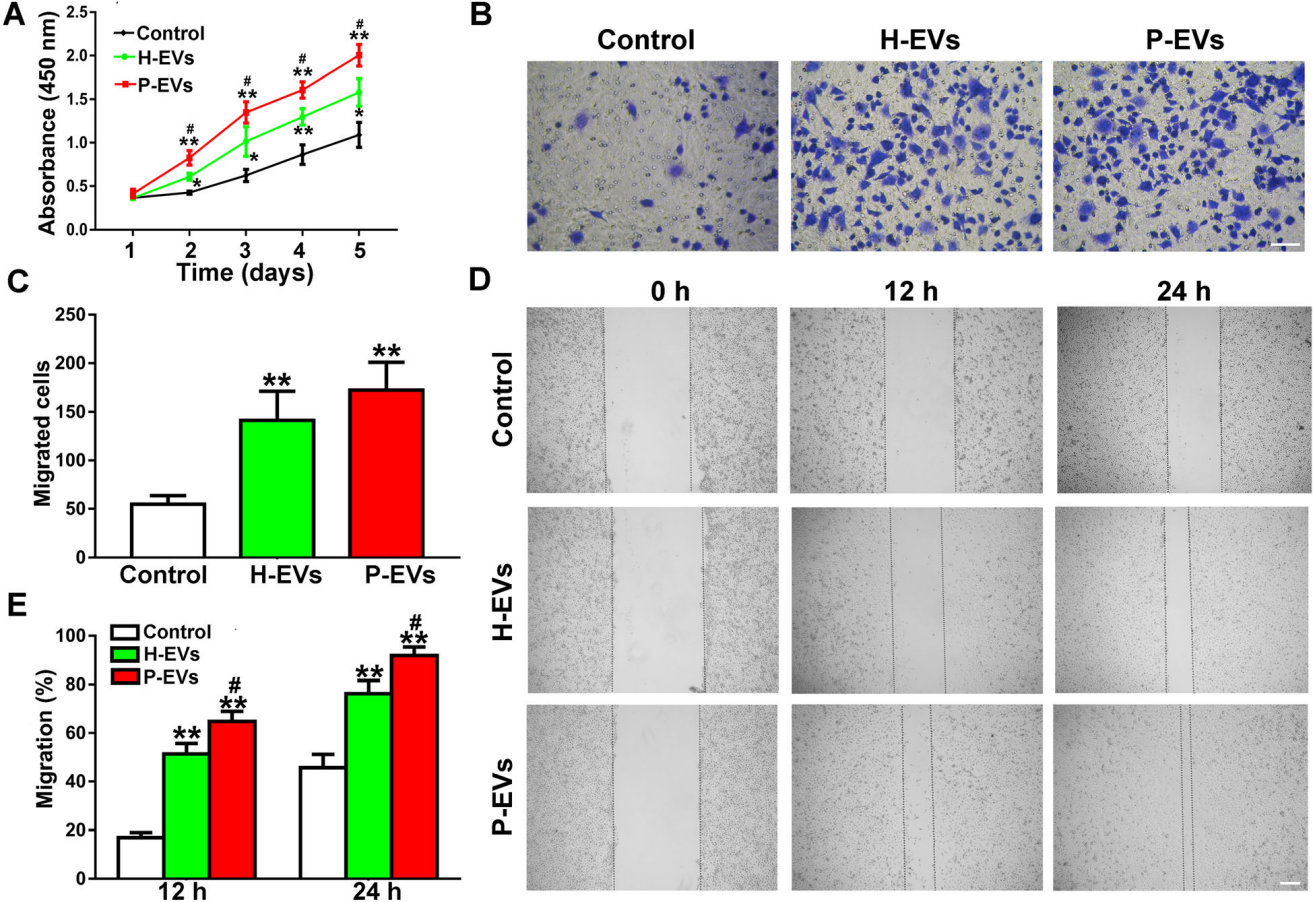
EVs enhanced the proliferation and migration of ECs in vitro. **a** The proliferation of ECs exposed to H-EVs, P-EVs, and an equal volume of PBS was tested by the CCK-8 assay (*n* = 3). **b** The migration of ECs stimulated by H-EVs, P-EVs, and PBS was detected by transwell assay (scale bar, 100 μm). **c** Quantitative analysis of the migrated cells in **b** (*n* = 5). **d** Representative images of the scratch wound assay in ECs treated with H-EVs, P-EVs, and PBS (scale bar, 200 μm). **e** Quantitative analysis of the migration rates in **d** (*n* = 3). *P < 0.05, **P < 0.01 vs. the control group; ^**#**^*P* < 0.05 vs. the H-EV group

ECs were treated with P-EVs, H-EVs, or an equal volume of PBS for a sequence of functional assays, including proliferation and migration, which are two critical steps of angiogenesis. The results obtained from the CCK-8 assay showed that the proliferation rate of ECs was markedly elevated in response to EV stimulation relative to the control group (*P* < 0.05 or *P* < 0.01; Fig. 5a), and notably, ECs exhibited a stronger proliferative rate by P-EVs than H-EVs (*P* < 0.05; Fig. 5a). The migration-promoting effect was determined by a scratch wound healing assay and a transwell assay. The results obtained from the transwell assay revealed that there was no difference between the two EV groups (*P* > 0.05; Fig. 5b, c), although both of them demonstrated a stronger migration-promoting ability than the control group (*P* < 0.01; Fig. 5b, c). In the scratch wound healing assay, both H-EVs and P-EVs remarkably upregulated the motility of ECs compared with that in the control group (*P* < 0.01; Fig. 5d, e), and P-EVs exhibited a stronger migration ability than H-EVs (*P* < 0.05; Fig. 5d, e).

#### P-EVs potentiated the angiogenic activities of ECs in vitro

**Fig. 6:**
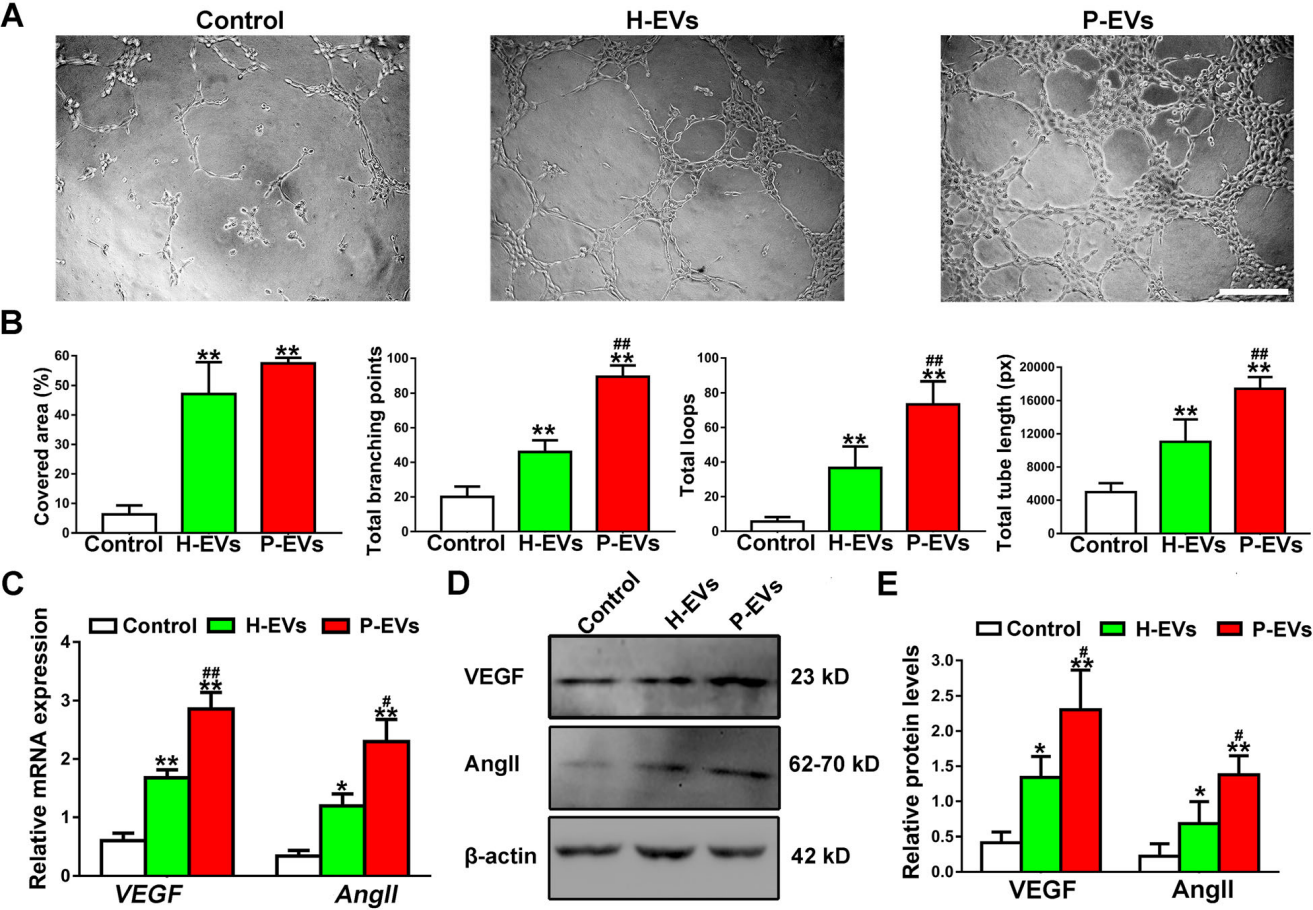
EVs potentiated the angiogenic activities of ECs in vitro. **a** Representative images of the tube formation assay in ECs treated with H-EVs, P-EVs, and an equal volume of PBS (scale bar, 200 μm). **b** Quantitative analyses of the covered area, total branching points, total loops, and total tube length in **b** (*n* = 3). **c** The proangiogenic genes *VEGF* and *AngII* in ECs were analyzed by qRT-PCR analysis (*n* = 3). **d** Detection of the protein levels of VEGF and AngII in ECs by western blot analysis. **e** Quantitative analysis of the relative protein expression (*n* = 3). *P < 0.05, **P < 0.01 vs. the control group; ^**#**^*P* < 0.05, ^**##**^*P* < 0.01 vs. the H-EV group

Furthermore, we evaluated the effect of EVs on angiogenic activities of ECs. Compared with the control group, both EV treatments significantly enhanced the angiogenic activities of ECs (*P* < 0.01; Fig. 6a, b). Moreover, compared with those treated with H-EVs, ECs treated with P-EVs showed a higher number of capillary-like structures, and quantitative measurements revealed that the total branching points, total loop numbers, and total tube length were all significantly increased (*P* < 0.01; Fig. 6b), although the covered area was not (*P* > 0.05; Fig. 6b). qRT-PCR and western blot analysis were performed to detect the expression levels of angiogenesis-related genes and proteins (VEGF and AngII). The obtained results demonstrated that both groups of EVs exhibited upregulated angiogenesis-related gene expression compared with that in the control group (*P* < 0.05 or *P* < 0.01; Fig. 6c); moreover, gene expression in the P-EV group was higher than that in the H-EV group (*P* < 0.05 or *P* < 0.01; Fig. 6c). Furthermore, ECs in the P-EV group secreted more VEGF and AngII proteins than ECs in the H-EV and control groups (*P* < 0.05 or *P* < 0.01; Fig. 6d, e).

### Effects of H-EVs and P-EVs on wound healing

#### P-EVs accelerated cutaneous wound healing in mice

**Fig. 7:**
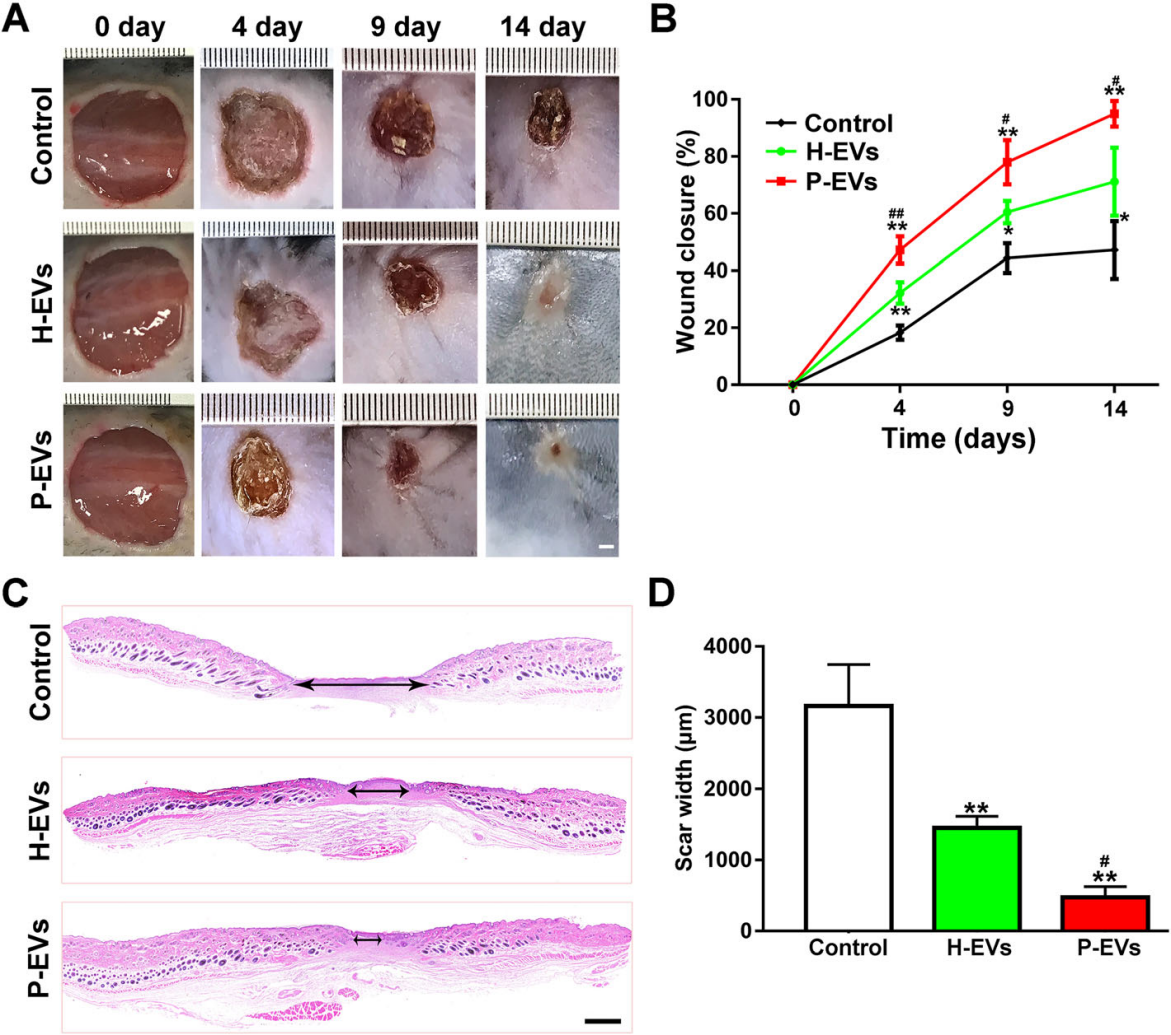
EVs accelerated cutaneous wound healing in mice. **a** Gross view of wounds treated with H-EVs, P-EVs, and PBS at days 4, 9, and 14 postwounding (scale bar, 1.5 mm). **b** The rate of wound closure in wounds receiving different treatments at the indicated times (*n* = 10). **c** H&E staining of wound sections treated with H-EVs, P-EVs, and PBS at 14 days after operation. The double-headed black arrows indicate the edges of the scars (scale bar, 500 μm). **d** Quantitative analysis of scar widths in **c** (*n* = 8). *P < 0.05, **P < 0.01 vs. the control group; ^**#**^*P* < 0.05, ^**##**^*P* < 0.01 vs. the H-EV group

A rat full-thickness defect model was created to assess the effects of EVs on wound healing. All the mice were maintained under specific pathogen-free (SPF) conditions, and none of the mice showed any signs of discomfort/disability following EV administration in the present study. Wound closure was markedly accelerated by EV treatment, illustrated by smaller wound areas measured at days 3, 9, and 14 postwounding compared with those in the control group (*P* < 0.05 or *P* < 0.01; Fig. 7a, b), and as expected, the rate of wound closure in the P-EV group was faster than that in the H-EV group (*P* < 0.05 or *P* < 0.01; Fig. 7b). In addition, the wounds treated with P-EVs had almost entirely closed by day 14 after the operation, whereas relatively larger scar areas remained detectable in the control group (Fig. 7a). H&E staining revealed that transplantation with both types of EVs reduced the scar formation of wounds compared with the control group at day 14 after treatment (*P* < 0.01; Fig. 7c, d), and quantitative measurements confirmed that P-EV-treated wounds had a lower level of scar formation than H-EV-treated wounds (*P* < 0.05; Fig. 7d).

#### P-EVs enhanced vessel formation in the wound sites of mice

**Fig. 8:**
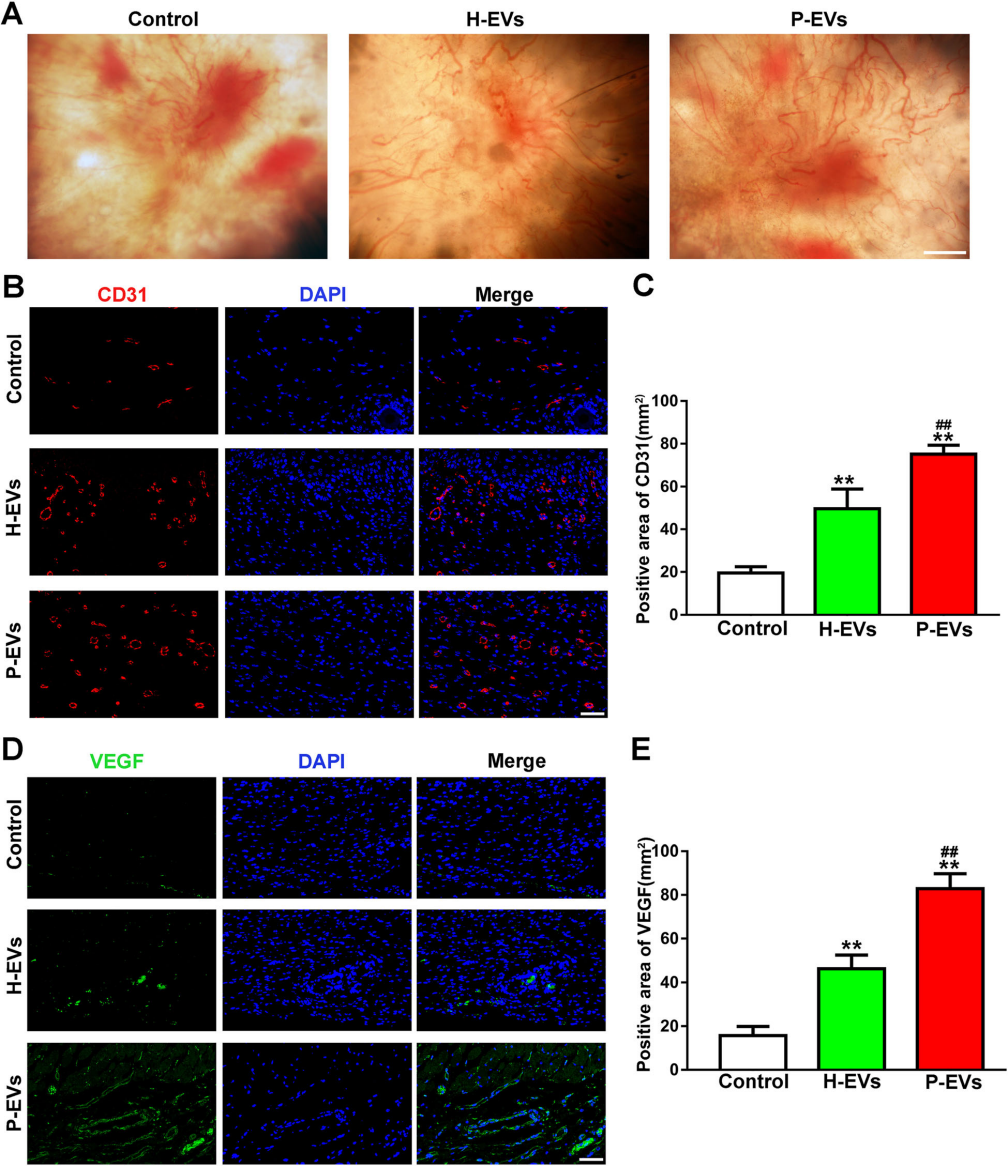
EVs enhanced angiogenesis at the mouse wound sites. **a** Gross view of the undersurface of wounds treated with H-EVs, P-EVs, and PBS at day 14 postwounding. Newly formed blood vessels were detected in the wound sites (scale bar, 1 mm). **b** CD31 immunofluorescence staining for wound sections treated with H-EVs, P-EVs, and PBS at day 14 postwounding (scale bar, 50 μm). **c** Quantitative analysis of the CD31-positive area in **b** (*n* = 8). **d** Representative images of VEGF staining for wound sections treated with H-EVs, P-EVs, and PBS at day 14 postwounding (scale bar, 50 μm). **e** Quantitative analysis of the VEGF-positive area in **d** (*n* = 8). **P < 0.01 vs. the control group; ^**##**^*P* < 0.01 vs. the H-EV group

Skin images from the undersurface revealed that both EV-treated groups exhibited more newly formed microvessels than control wounds at day 14 postwounding (Fig. 8a). Immunofluorescence staining for endothelial markers, including CD31 and VEGF, was also performed to quantify the density of new blood vessels. Representative images of CD31 staining consistently demonstrated that abundant blood vessels appeared in the wounds treated with EVs, whereas the capillary density was significantly lower in the control group (*P* < 0.01; Fig. 8b, c). Quantitative analysis revealed that the proangiogenic effect of P-EVs was stronger than that of H-EVs (*P* < 0.01; Fig. 8c). Similarly, more extensive blood vessel staining for VEGF was observed in the P-EV group than in the H-EV and control groups (*P* < 0.01, Fig. 8d, e).

## Discussion

The regeneration of dental pulp tissue must incorporate a bioengineering strategy to promote cell chemotaxis and angiogenesis and, hence, fast vascularization of newly formed tissue [29]. Only when new vessels and vasculature can provide oxygen and nutrients to the stem cells involved in the reparative process can functional tissue regeneration be finally successful. However, the ability of current strategies to promote angiogenesis/vasculogenesis to form vascularized dental pulp tissue is critically limited [30]. Given the potential of DPSCs to differentiate into ECs and to promote angiogenesis, current bioengineering approaches have focused on the use of those cells for dental pulp tissue engineering with the aim of regenerating vascularized pulp tissues [31–33]. As a more readily available cell source in the clinic, P-DPSCs exhibit similar regenerative potential to H-DPSCs and have also been shown to give rise to functional pulp regeneration [11–15]. However, the proangiogenic capacity of P-DPSCs has not yet been described. This is the first study to explore the role of P-DPSCs in angiogenesis/vasculogenesis, and our findings will help pave the way to using this easily assessable cell population in dental pulp tissue engineering.

With in-depth studies of MSCs, growing evidence has demonstrated that the therapeutic effects of MSCs contributing to angiogenesis and tissue repair are predominantly attributed to paracrine function [34]. These paracrine secretions include a variety of soluble factors and EVs, which regulate repair and regeneration processes by affecting the activity of resident cells [35, 36]. EVs, in particular, have been confirmed as the principal factor contributing to the paracrine function-mediated therapeutic efficacy of donor cells [37]. These nanosized EVs are lipid bilayer structures containing proteins, nucleic acids, and lipids that can contribute to intercellular communications by transferring these specific molecules, further exerting biological effects [38, 39]. In fact, EVs are attractive as therapeutics due to their cellular-related advantages, such as high stability, no risk of aneuploidy, and low propensity to trigger immune rejection following allogeneic administration [40–42]. In this context, an increasing number of studies have demonstrated that EVs derived from various types of stem cells have therapeutic potential for angiogenesis [43–45]. More importantly, EVs derived from normal DPSCs have been demonstrated to trigger regeneration of dental pulp-like tissue [18] and indeed exhibit the capacity to promote angiogenesis in vitro and in vivo [19]. Therefore, the secreted elements contained within CMs derived from P-DPSCs, particularly their secreted EVs, were applied to investigate the proangiogenic effects of P-DPSCs.

In this particular study, patient-matched H-DPSCs/EVs were used as the control for P-DPSCs/EVs to eliminate the interference of some confounding factors (e.g., age and gender). We confirmed the proangiogenic effects of P-EVs and, for the first time, to the best of our knowledge, compared their proangiogenic potential with that of H-EVs. Although both H-EVs and P-EVs were found to enhance the angiogenesis-related activities of ECs, P-EVs exerted a more robust potential to stimulate EC proliferation, migration, and tube formation (Figs. 5 and 6). Additionally, a recent study demonstrated that EVs derived from lipopolysaccharide (LPS)-preconditioned DPSCs had a better ability to promote Schwann cell proliferation, migration, and odontogenic differentiation than EVs from normal DPSCs [46]. In addition, MSCs were reported to secrete more EVs under inflammatory conditions than under normal conditions [47]. In line with this, P-DPSCs secreted more EVs than H-DPSCs (P-EVs vs. H-EVs, 1.73 × 10^9^ vs. 5.67 × 10^8^ particles/mL) in the present study. When a full-thickness skin defect model of C57BL/6 mice was applied to explore the effects of EVs on angiogenesis, both P-EVs and H-EVs were found to accelerate wound healing and promote vascularization across skin defects in mice, but wounds treated with P-EVs resulted in a quicker healing outcome and led to more new vessel formation (Figs. 7 and 8). These encouraging results collectively suggest that P-EVs possess a relatively higher proangiogenic ability than H-EVs. As reported previously, a full-thickness skin defect in mice is a valid animal model to investigate the initial proangiogenic effects of cellular materials in terms of vascularization and new vessel formation [21, 23]. However, the potential use of P-DPSCs/P-EVs toward regenerating vascularized pulpal tissues must be tested in relevant pulp insult animal models.

Although our findings suggest that the inflammatory microenvironment in periodontitis does not negatively affect the proangiogenic effects of P-DPSCs, those cells were found to express lower levels of CD146 than H-DPSCs isolated from the pulp tissue of the same donor (Fig. 1), suggesting that the cells must have undergone some changes in response to inflammation [48, 49]. Indeed, the periodontium closely connects to the pulp-dentin complex by three interconnected routes, namely, the apical foramen, the dentinal tubules, and the lateral canals, which means that once periodontitis involves a certain tooth, the dental pulp may be negatively affected by bacteria, especially by their byproducts residing in the periodontitis pocket [50, 51]. For example, LPS is produced by gram-negative bacteria, which can induce various types of proinflammatory cytokines and chemokines such as IL-1, IL-6, and IL-8 as well as tumor necrosis factor alpha (TNF-a); these proinflammatory cytokines may affect a series of biological processes such as survival, proliferation, and differentiation of MSCs [11, 52–56]. In addition, the pulp is tightly enclosed by dentin (hard tissues), and the apical foramen is almost the only root to supply blood for pulp tissues. Therefore, if periodontal inflammation cannot be arrested, combined with poor blood flow, the dental pulp will inevitably undergo necrosis [52]. However, in our previous study, it was demonstrated that although P-DPSCs suffer relatively long-term stimulation by inflammation, regenerative-related properties such as osteoblastic differentiation might increase to some extent, as long as the vitality of the pulp has not been totally damaged [14]. Herein, in the present study, we ruled out teeth with completely necrotized pulp tissue before pulling them out. As long as the pulp is still viable, to counteract the inflammatory invasion, P-DPSCs may have a specific capacity for the production of proangiogenic molecules, such as VEGF, because angiogenesis could be induced by inflammation [11, 52, 57]. Generally, the formation of new blood vessels is in direct response to tissue demands [58]. Thus, P-DPSCs in turn strongly produce VEGF in response to diverse stimulants, and new vessels appear in the inflammatory sites of dental pulp to compensate for the insufficient blood supply in the pulp cavity.

MSC-derived EVs can load certain molecules that recapitulate the biological effects of parent cells [59]. Indeed, EVs from different stem cells possess innate differences, further leading to diverse biological functions, as they faithfully reflect the genomic characteristics of their source cells [40]. Thus, P-EVs may contain certain proangiogenic molecules that can mediate angiogenesis activities. However, the underlying mechanisms by which P-EVs promote angiogenesis remain unclear. It has been reported that EVs derived from MSCs contain various proteins and microRNAs that can mediate the bioactivity of target cells via activation of certain signaling pathways as well as regulation of related protein translation [60, 61]. Thus, in the next study, we will compare the composition of biomolecules (such as common or cell-specific microRNA) in P-EVs and H-EVs to explore the underlying mechanisms by which P-EVs enhance angiogenic activities. Such investigations may provide more specific information to help identify the effect of periodontitis on pulpal property changes and the proper use of P-DPSCs in regenerative medicine.

## Conclusions

Based on a comparison with patient-matched H-DPSCs, this is the first endeavor to describe the proangiogenic potential of P-DPSCs with an emphasis on their secreted EVs. Data obtained from this study showed that P-EVs derived from P-DPSCs could profoundly enhance the proliferation, migration, and angiogenesis of ECs in vitro and were able to accelerate cutaneous wound healing and promote vessel formation in vivo. Our findings suggest that the use of P-DPSCs can facilitate new vessel formation in cellular therapy and regenerative medicine. More importantly, our data reveal that although the inflammatory microenvironment across the periodontium can negatively influence the living condition of dental pulp and, hence, impair the pluripotential capacity of residing DPSCs, the proangiogenic potential of those cells is not affected. In contrast, living in an environment with the presence of various proinflammatory cytokines (e.g., slightly inflamed pulp tissue as a result of periodontitis) may enhance the proangiogenic potential of P-DPSCs. Although the present study further explores the potential to use P-DPSCs in dental pulp tissue engineering, the molecular mechanism underlying the enhanced proangiogenic effect of those cells remains unknown. In particular, the signaling pathways involved in EVs derived from P-DPSCs during angiogenesis/vasculogenesis warrant further investigation.

## Supplementary information

**Additional file 1: Figure S1.** Isolation of H-DPSCs and I-DPSCs. (A) Representative images of primary cells derived from human pulp tissue within periodontally healthy teeth (upper) and periodontally compromised teeth (lower) at day 7 (scale bar: 500 μm). (B) Morphologic appearance of H-DPSCs and P-DPSCs observed by an inverted microscope (Scale bar: 500 μm).

**Additional file 2: Figure S2.** GW4869 inhibited EV secretion without influencing cell growth. DPSCs were treated for 12 h with GW4869 (10 μM) or DMSO. Then, the cells were washed with PBS and cultured with serum-free α-MEM media. (A) After further incubation for 48 h, the number of cells was counted at the end of EV production. (B) The EVs were purified, and the total protein of EVs was detected (*n* = 3). *P < 0.01 vs. DMSO group.

## Data Availability

All data generated or analyzed during this study are included in this published article.

## Abbreviations

MSC: Mesenchymal stem cell
DPSCs: Dental pulp stem cells
H-DPSCs: Dental pulp stem cells within healthy teeth
P-DPSCs: Dental pulp stem cells from teeth extracted due to periodontitis
ECs: Endothelial cells
CM: Conditioned medium
EV: Extracellular vesicle
H-EVs: Extracellular vesicles from dental pulp stem cells within healthy teeth
P-EVs: Extracellular vesicles from dental pulp stem cells within teeth extracted due to periodontitis
CCK-8: Cell Counting Kit-8
EGM-2: Endothelial growth medium-2
qRT-PCR: Quantitative reverse transcriptase-polymerase chain reaction
H&E: Hematoxylin and eosin
