# Correction: The proangiogenic effects of extracellular vesicles secreted by dental pulp stem cells derived from periodontally compromised teeth

**DOI:** 10.1186/s13287-022-03192-5

**Published:** 2022-10-24

**Authors:** Huan Zhou, Xuan Li, Yuan Yin, Xiao-Tao He, Ying An, Bei-Min Tian, Yong-Long Hong, Li-An Wu, Fa-Ming Chen

**Affiliations:** 1 State Key Laboratory of Military Stomatology, National Clinical Research Center for Oral Diseases, School of Stomatology, Fourth Military Medical University, Xi’an, 710032 Shaanxi People’s Republic of China; 2 Stomatology Center, Shenzhen Hospital of Southern Medical University, Shenzhen, Guangdong People’s Republic of China

## Correction to: Stem Cell Research & Therapy (2020) 11:110 https://doi.org/10.1186/s13287-020-01614-w

Following the publication of this article [1], the authors notice that the article included misused or duplicated elements.

Figure 2D, the labels of the two groups (H-CM and P-CM) were wrongly set, which should be interchanged. Besides, the scratch wound assay image of the middle panel for 24 h P-CM in Fig. 2D (indeed should be H-CM) was duplicated with the top panel for 24 h H-CM in Fig. 3D.

**Fig. 2:**
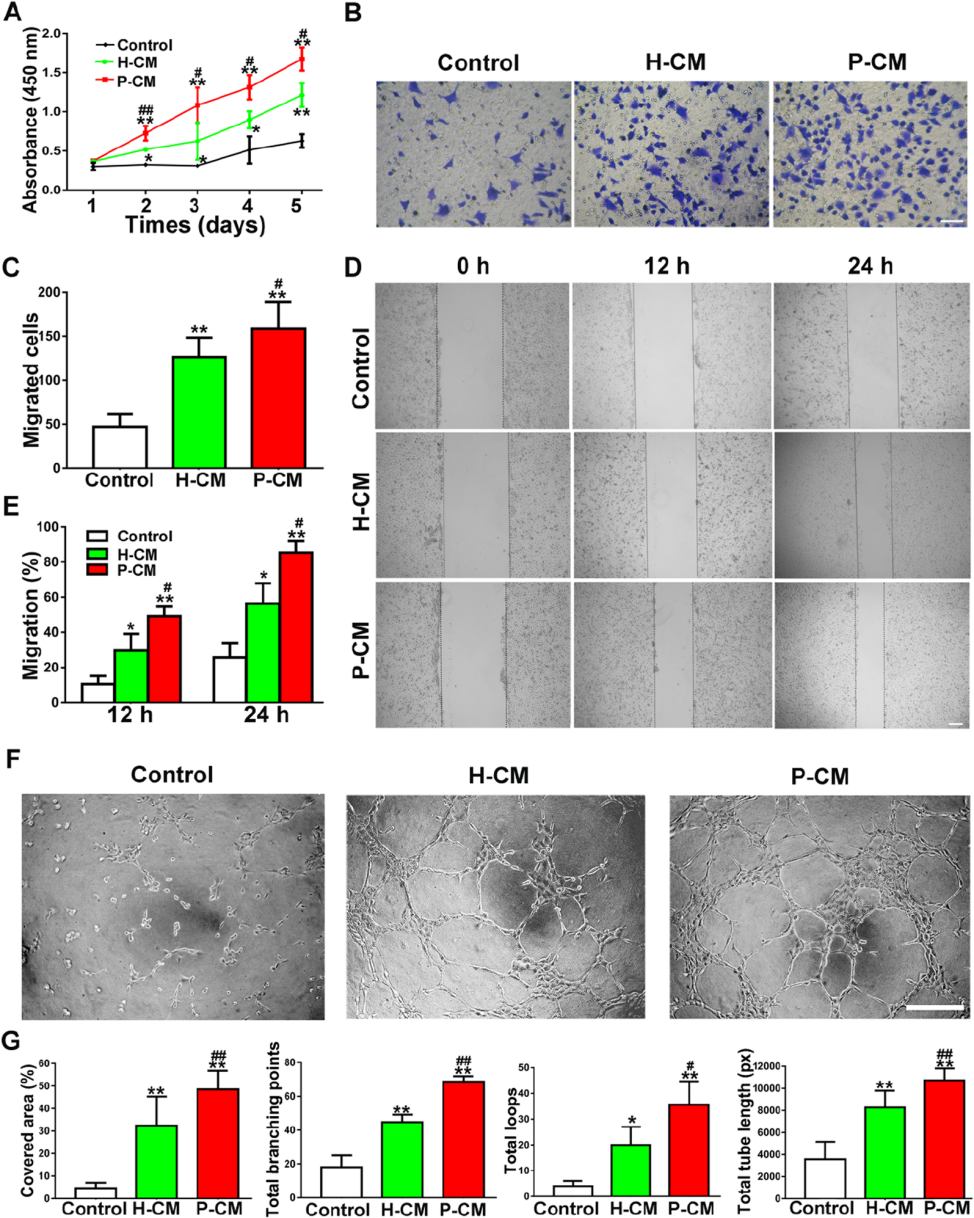
H-CM and P-CM enhanced the angiogenic activities of ECs. **a** The proliferation of ECs exposed to H-CM and P-CM was tested by CCK-8 assay (*n* = 3). **b** The migration of ECs stimulated by H-CM and P-CM was detected by transwell assay (scale bar, 100 μm). **c** Quantitative analysis of the migrated cells in **b** (*n* = 5). **d** Representative images of the scratch wound assay of ECs treated with H-CM and P-CM (scale bar, 200 μm). **e** Quantitative analysis of the migration rates in **d** (*n* = 5). **f** Representative images of the tube formation assay in ECs treated with H-CM and P-CM (scale bar, 200 μm). **g** Quantitative analyses of the covered area, total branching points, total loops, and total tube length in **f** (*n* = 3). *P < 0.05, **P < 0.01 versus the control group; ^#^*P* < 0.05, ^##^*P* < 0.01 versus the H-CM group

Figure 8A: there were overlapped parts for gross view of the undersurface of wounds in Control group and P-EVs group.

**Fig. 8:**
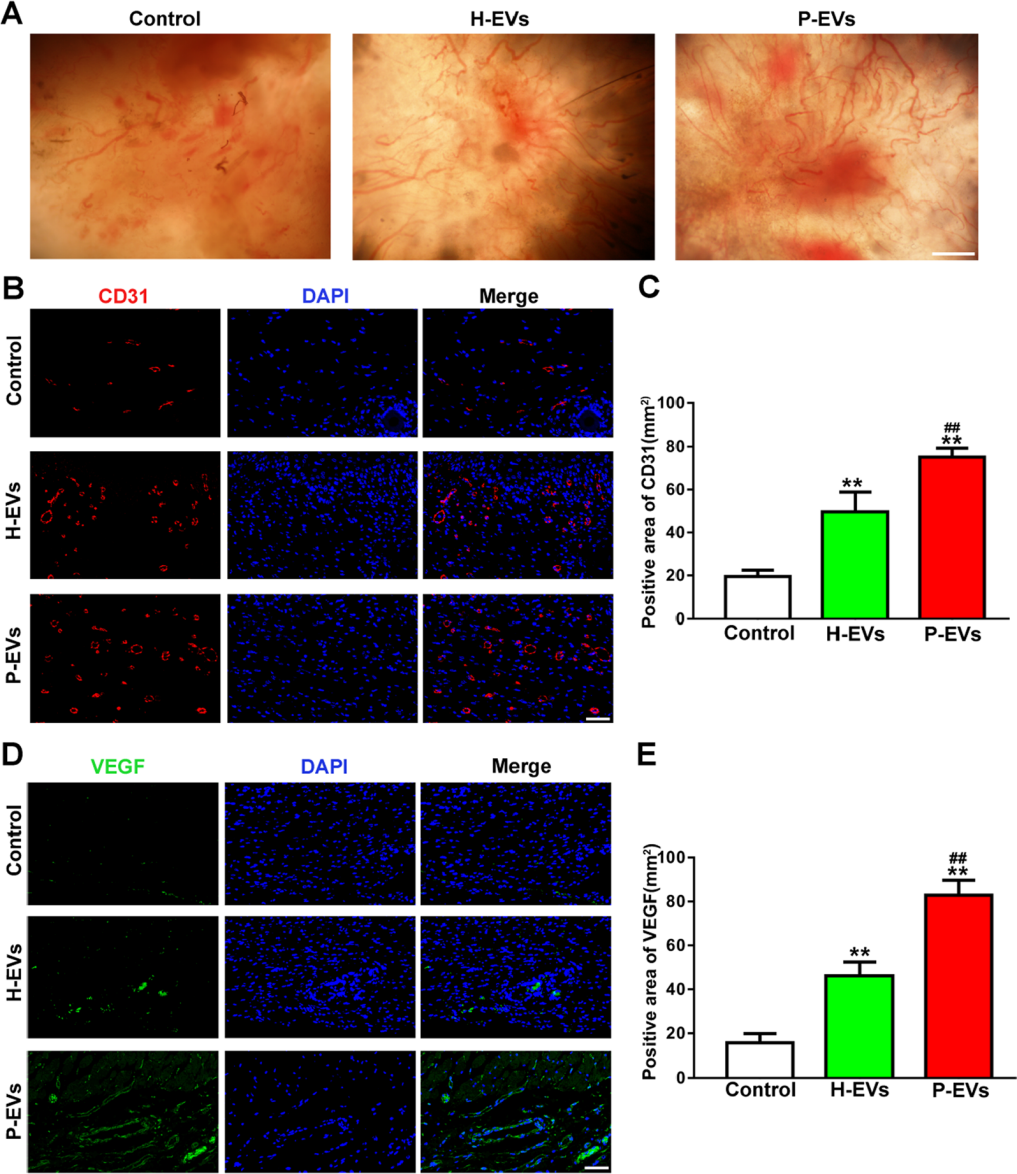
EVs enhanced angiogenesis at the mouse wound sites. **a** Gross view of the undersurface of wounds treated with H-EVs, P-EVs, and PBS at day 14 postwounding. Newly formed blood vessels were detected in the wound sites (scale bar, 1 mm). **b** CD31 immunofluorescence staining for wound sections treated with H-EVs, P-EVs, and PBS at day 14 postwounding (scale bar, 50 μm). **c** Quantitative analysis of the CD31-positive area in **b** (*n* = 8). **d** Representative images of VEGF staining for wound sections treated with H-EVs, P-EVs, and PBS at day 14 postwounding (scale bar, 50 μm). **e** Quantitative analysis of the VEGF-positive area in **d** (*n* = 8). **P < 0.01 versus the control group; ^##^*P* < 0.01 versus the H-EV group

After checking the original data, the authors realized that there were errors with the image selection during manuscript preparation. The corrected figures (Figs. 2, 8) along with their corresponding captions are listed below.

In addition, in Section “Mouse skin wound model and treatments”, the statement “Thirty male C57BL/6 mice (8 weeks old, weighing 20–25 g; purchased from the Animal Research Committee of FMMU) were used in this study, and all procedures were approved by the Animal Research Committee of FMMU” at the beginning of this Section was wrong. In fact, the mice were purchased from the Laboratory Animal Center of FMMU instead of the Animal Research Committee of FMMU.

The correction will not affect the finding and conclusion of the manuscript. The authors regret for the inadvertent errors during manuscript preparation and apologize for any inconvenience caused.
