# Supplementary material for: The proangiogenic effects of extracellular vesicles secreted by dental pulp stem cells derived from periodontally compromised teeth
Source: Stem Cell Res Ther. 2020 Mar 6;11:110. doi: 10.1186/s13287-020-01614-w (PMC7060605; doi:10.1186/s13287-020-01614-w)
Supplement: Supplementary file 1 — Additional file 1: Figure S1. Isolation of H-DPSCs and I-DPSCs. (A) Representative images of primary cells derived from human pulp tissue within periodontally healthy teeth (upper) and periodontally compromised teeth (lower) at day 7 (scale bar: 500 μm). (B) Morphologic appearance of H-DPSCs and P-DPSCs observed by an inverted microscope (Scale bar: 500 μm). [file 13287_2020_1614_MOESM1_ESM.docx]

**Additional material**

**Fig. S1.**

**
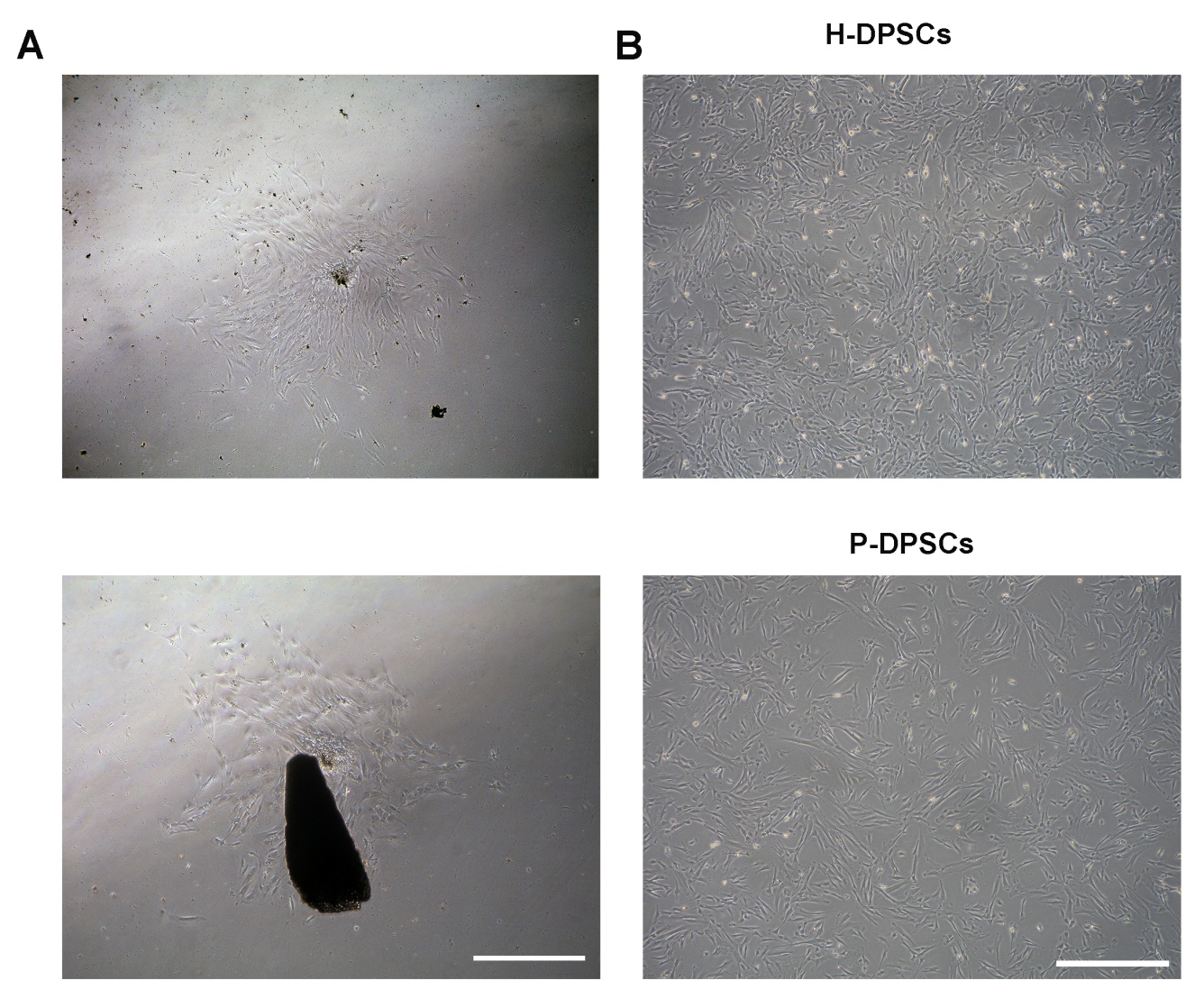
**

**Fig. S1. Isolation of H-DPSCs and I-DPSCs.** (A) Representative images of primary cells derived from human pulp tissue within periodontally healthy teeth (upper) and periodontally compromised teeth (lower) at day 7 (scale bar: 500 μm). (B) Morphologic appearance of H-DPSCs and P-DPSCs observed by an inverted microscope (Scale bar: 500 μm).
