# Supplementary material for: The proangiogenic effects of extracellular vesicles secreted by dental pulp stem cells derived from periodontally compromised teeth
Source: Stem Cell Res Ther. 2020 Mar 6;11:110. doi: 10.1186/s13287-020-01614-w (PMC7060605; doi:10.1186/s13287-020-01614-w)
Supplement: Supplementary file 2 — Additional file 2: Figure S2. GW4869 inhibited EV secretion without influencing cell growth. DPSCs were treated for 12 h with GW4869 (10 μM) or DMSO. Then, the cells were washed with PBS and cultured with serum-free α-MEM media. (A) After further incubation for 48 h, the number of cells was counted at the end of EV production. (B) The EVs were purified, and the total protein of EVs was detected (n = 3). *P < 0.01 vs. DMSO group. [file 13287_2020_1614_MOESM2_ESM.docx]

**Additional material**

**Fig. S2.**


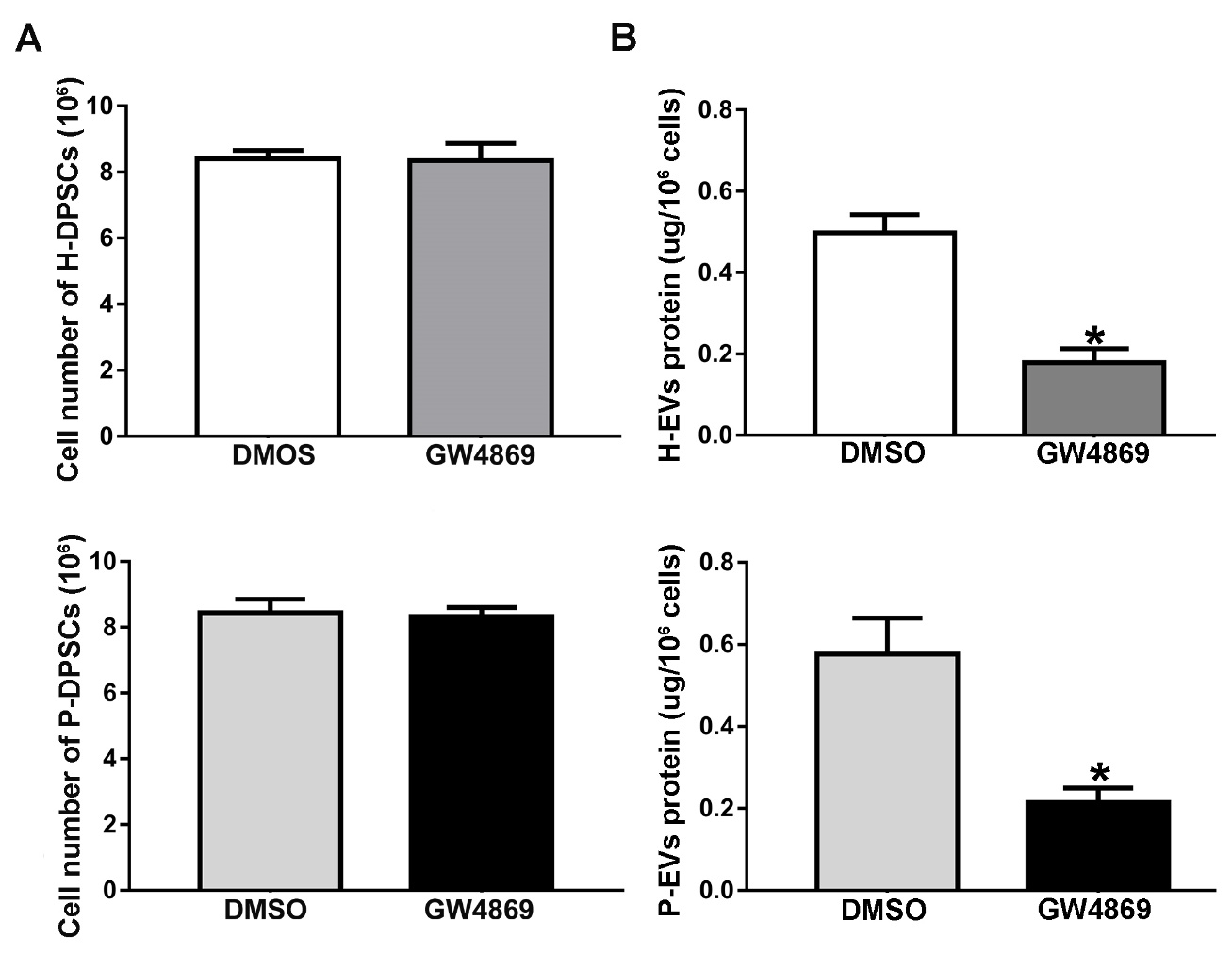


**Fig. S2. GW4869 inhibited EV secretion without inﬂuencing cell growth.** DPSCs were treated for 12 h with GW4869 (10 μM) or DMSO. Then, the cells were washed with PBS and cultured with serum-free α-MEM media. (A) After further incubation for 48 h, the number of cells was counted at the end of EV production. (B) The EVs were puriﬁed, and the total protein of EVs was detected (*n* = 3). **P* < 0.01 vs. DMSO group.
